# Toward monochromated sub-nanometer UEM and femtosecond UED

**DOI:** 10.1038/s41598-020-73168-z

**Published:** 2020-09-30

**Authors:** Xi Yang, Weishi Wan, Lijun Wu, Victor Smaluk, Timur Shaftan, Yimei Zhu

**Affiliations:** 1 National Synchrotron Light Source II, Brookhaven National Laboratory, Upton, NY 11973 USA; 2 School of Physical Science and Technology, ShanghaiTech University, Shanghai, China; 3 Condensed Matter Physics and Materials Science Division, Brookhaven National Laboratory, Upton, NY 11973 USA

**Keywords:** Condensed-matter physics, Particle physics, Techniques and instrumentation, Physics, Imaging techniques

## Abstract

A preliminary design of a mega-electron-volt (MeV) monochromator with 10^−5^ energy spread for ultrafast electron diffraction (UED) and ultrafast electron microscopy (UEM) is presented. Such a narrow energy spread is advantageous in both the single shot mode, where the momentum resolution in diffraction is improved, and the accumulation mode, where shot-to-shot energy jitter is reduced. In the single-shot mode, we numerically optimized the monochromator efficiency up to 13% achieving 1.3 million electrons per pulse. In the accumulation mode, to mitigate the efficiency degradation caused by the shot-to-shot energy jitter, an optimized gun phase yields only a mild reduction of the single-shot efficiency, therefore the number of accumulated electrons nearly proportional to the repetition rate. Inspired by the recent work of Qi et al. (Phys Rev Lett 124:134803, 2020), a novel concept of applying reverse bending magnets to adjust the energy-dependent path length difference has been successfully realized in designing a MeV monochromator to achieve the minimum energy-dependent path length difference between cathode and sample. Thanks to the achromat design, the pulse length of the electron bunches and the energy-dependent timing jitter can be greatly reduced to the 10 fs level. The introduction of such a monochromator provides a major step forward, towards constructing a UEM with sub-nm resolution and a UED with ten-femtosecond temporal resolution. The one-to-one mapping between the electron beam parameter and the diffraction peak broadening enables a real-time nondestructive diagnosis of the beam energy spread and divergence. The tunable electric–magnetic monochromator allows the scanning of the electron beam energy with a 10^−5^ precision, enabling online energy matching for the UEM, on-momentum flux maximizing for the UED and real-time energy measuring for energy-loss spectroscopy. A combination of the monochromator and a downstream chicane enables “two-color” double pulses with femtosecond duration and the tunable delay in the range of 10 to 160 fs, which can potentially provide an unprecedented femtosecond time resolution for time resolved UED.

## Introduction

MeV UED and UEM provide a unique opportunity of simultaneous high temporal and spatial resolution for time-resolved observations and measurements in physics, chemistry and biology^1–12^. Examples include visualizing structure dynamics of aperiodic materials such as proteins that cannot be crystallized^13^. By employing an accelerator-based radiofrequency (RF) photoinjector as the MeV electron source, UED/UEM takes advantage of the strong interaction between electrons and matter while minimizing the space charge problem. Due to the two orders of magnitude shorter wavelength of electrons compared to X-rays allowing access to high scattering vectors in momentum space, UED/UEM can potentially resolve much finer structural details enabling us soon to see how atoms move in crystals and make molecular movies of chemical reactions. Together with XFEL these ultrafast probes will provide a more complete picture in groundbreaking studies of all kinds of complex dynamic processes in nature^5^.

Of the many technical challenges that are preventing MeV UED and UEM from reaching their full potential as significant tools in ultrafast science and technology, large energy spread of the electron source based on the RF-photocathode is at the top of the list. For example, the simulation carried out using Impact-T code^14^, which takes the space-charge effects into proper consideration, shows the energy spread of the electron beam increased significantly (from $1.5 \cdot 10^{ - 3}$ to $1.3 \cdot 10^{ - 2}$.) with the beam charge increasing from 1 to 13 pC. The beam energy spread and angular divergence are the main factors determining the diffraction peak width^10^. The momentum resolution of lens-less imaging in the reciprocal lattice depends on the interference between the probe and the object which requires very high beam monochromaticity. The Bragg diffraction (BD) angle is defined as the BD peak position on the detector in a far-field diffraction setup. Compared to the BD angle from an XFEL pulse, the Bragg angle diffracted by a MeV electron pulse is normally one-to-two orders magnitude smaller, in the range of one to several milliradians, due to the significantly shorter De Broglie wavelength of a MeV electron beam. To achieve a similar angular resolution required by structural dynamics (typically in the range from $10^{ - 3}$ to $10^{ - 2}$ for many metals and oxides)^15–24^, the BD peak width has to be minimized via reducing the energy spread and divergence of an electron beam. The independent control of the divergence as well as the size of the electron beam in the charge range from sub-pC to 13 pC has been experimentally demonstrated^10^. We will focus on the control of the energy spread and shot-to-shot energy jitter of the electron beam in the paper.

The design of an imaging lens system in a compact MeV UEM for single- and multi-shot imagining with sub-nm resolution has been reported^12^. To achieve this ambitious design goal, it is desirable to correct the intrinsic chromatic aberration of the RF-based UEM system, which is technically very challenging^25^. To reach the 0.4 nm spatial resolution of the 4 MeV UEM^12^ without aberration correction, the energy spread of the electron beam must be $\le \;10^{ - 5}$ in the single-shot mode, and both the shot-to-shot energy jitter and the single-shot energy spread must be $\le \;10^{ - 5}$ in the accumulation mode. At the same time, high-quality imaging requires the electron flux higher than 10^6^ per pulse, depending on the detector parameters such as the minimum number of electrons per pixel determined by the signal-to-noise threshold, the total number of pixels. It is difficult to achieve the above requirements even with the state-of-art photo-cathode RF electron gun. Both the single-shot energy spread and the shot-to-shot energy jitter need further improvement.

An alternative approach satisfying these stringent requirements is to implement a narrow-band energy filter, named monochromator, which passes through electrons only within a narrow energy bandwidth, e.g. $\Delta E{/}E \approx 10^{ - 5}$. This filtering process affects both the energy spread and the shot-to-shot energy jitter^26^. Although the concept presented in reference^27^ potentially can reach 100% efficiency with $10^{ - 7}$ level energy width, it has not been proven in practice and the use of time varying field makes synchronization very challenging. As a result, we decide to take the more traditional approach of using bending magnets with static field. To preserve the pulse length and reduce timing jitter, reverse bending magnets are included, making the monochromator a bunch compressor simultaneously. Besides, we improve the monochromator efficiency via maximizing the number of electrons *n* within the energy acceptance and the repetition rate *N* of the electron pulses. As a result, the electron flux having energies within the energy acceptance contributing to the diffraction signal is equal to *n*⋅*N*.

To enable UED as the probe in pump-probe experiments with an angular resolution $\Delta \theta {/}\theta$ comparable to XFEL (≤ 10^−3^)^10^, an aperture with both x and y limitations is placed where the maximum dispersion (*D*_max_) is with vanishing dispersion slope (*D*′ = 0)^26^. This aperture simultaneously controls the beam divergence $(\Delta \theta_{{e^{ - } }} \approx 10 \;\upmu {\text{rad}})$ and the energy resolution (${\Delta }E/E \le 10^{ - 5}$); together they determine the BD peak width via the equation $\sigma_{BD} \approx \sqrt {\left( {\frac{\Delta E}{E}\theta_{Bragg} } \right)^{2} + \Delta \theta_{{e^{ - } }}^{2} }$, where $\theta_{{{\text{Bragg}}}}$ is the Bragg angle. Furthermore, one can optimize the condenser lens to achieve a “pencil beam” with the minimum divergence at the sample. Considering the nominal Bragg angle of a few milliradian, the angular resolution can be 10^−3^ or better. The monochromator also allows the implementation of “two-color” double pulses with femtosecond duration and the tunable delay in the range of 10–160 fs via a double-slit configuration. This scheme can potentially provide femtosecond temporal resolution for the time resolved UED. Moreover, the “two-color” double-pulse scheme is easily expandable to “multi-color” multiple pulses. This makes the simultaneous high temporal and angular resolution of the UED comparable with XFELs (e.g. at LCLS). The angular resolution of $10^{ - 3}$ and the femtosecond temporal resolution provide the capability of probing a wide range of structural dynamics.

Furthermore, the electron energy scan with a narrow bandwidth can provide online energy matching for UEM and maximize the electron on-momentum flux for UED. Further improvement of the energy resolution may satisfy the requirement of energy loss measurement^28,29^.

## Results

### Design considerations of the monochromator

Designing an energy filter for UED and/or UEM we must consider the followings:

- To meet the design energy spread $\Delta E/E$ of 10^−5^ and the realistic aperture size *a* of several micrometers, the dispersion ($D_{x}$) is expected to be tens centimeter^26^.
- To maximize the monochromator efficiency via minimizing the transverse beam loss, the condition “$$\sigma_{\beta ,x} < \sigma_{D,x}$$” must be satisfied. The transverse beam size, shown as Eq. (1)^26^, is the quadratic sum of two parts—one from the transverse beam emittance $$\varepsilon_{x}$$ and beta function $$\beta_{x}$$ via ($$\sigma_{\beta ,x} = \sqrt {\beta_{x} \cdot \varepsilon_{x} }$$) and the other from the longitudinal beam energy spread $${\raise0.7ex\hbox{${\Delta E}$} \!\mathord{\left/ {\vphantom {{\Delta E} E}}\right.\kern-\nulldelimiterspace} \!\lower0.7ex\hbox{$E$}}$$ and dispersion $$D_{x}$$ via ($$\sigma_{D,x} = D_{x} \cdot {\raise0.7ex\hbox{${\Delta E}$} \!\mathord{\left/ {\vphantom {{\Delta E} E}}\right.\kern-\nulldelimiterspace} \!\lower0.7ex\hbox{$E$}}$$). Here, $$\sigma_{D,x} \approx 6.5\;\upmu {\text{m}}$$ is determined by the bandwidth of the monochromator $$(10^{ - 5}$$) and the dispersion at the aperture (0.645 m). Assuming a reasonable $$\varepsilon_{x} \approx 10\;{\text{ nm}} \cdot {\text{rad}}$$, “$$\sigma_{\beta ,x} < \sigma_{D,x}$$” puts a stringent constrain on the beta function $$\beta_{x} < 0.004\;{\text{m}}$$, therefore the beam losses mainly due to $${\raise0.7ex\hbox{${\Delta E}$} \!\mathord{\left/ {\vphantom {{\Delta E} E}}\right.\kern-\nulldelimiterspace} \!\lower0.7ex\hbox{$E$}}$$ exceeding the monochromator bandwidth. A significant amount of the design effort spent in minimizing the beta function to achieve $$\beta_{x} = 0.002\;{\text{m}}$$. The size of the aperture *a* is 7.9 µm.
  1 $$\sigma_{x} = a = \sqrt {\sigma_{\beta ,x}^{2} + \sigma_{D,x}^{2} } = \sqrt {\beta_{x} \cdot \varepsilon_{x} + \left( {D_{x} \cdot {\raise0.7ex\hbox{${\Delta E}$} \!\mathord{\left/ {\vphantom {{\Delta E} E}}\right.\kern-\nulldelimiterspace} \!\lower0.7ex\hbox{$E$}}} \right)^{2} }$$
- To minimize the timing jitter due to different beam energy, the quantity *R*_56_, which is defined as $\frac{{v_{0} dt}}{{d\left( {p/p_{0} } \right)}}$ where $v_{0} dt = dz$, is designed to match the energy chirp of the incoming electron beam^26^. Based on the work of Qi et al., instead of constructing a double bend achromat with three quadrupoles^30^, reverse bending magnets are placed in the monochromator to adjust the energy-dependent path length difference (*R*_56_) such that the overall energy-dependent path length difference between cathode and sample is minimized achieving the bunch compression. Due to the space-charge effect, electrons in the bunch tail have lower energies than electrons in the head; they go through shorter pathlengths in the monochromator catching up with electrons in the head. Thanks to the achromat design, all electrons with different energies arrive at the sample simultaneously. For the current design of the monochromator with $R_{56} = { }0.069{\text{ m}}$, the same order of magnitude for cancelling the bunch lengthening effect between the cathode and the sample, only three different types of dipole magnets are required; this greatly simplifies the manufacturing and commissioning processes. For a beam with the energy chirp different from the design value, coming to the monochromator designed for the “two-color” double-pulse scheme, a downstream chicane can be used to compensate the residual chirp, achieving achromaticity in a broad range of operation energy.
- To provide a flexible match between the optimization via General Particle Tracer (GPT) simulation^31^, aiming for the minimum energy spread of the electron beam after the gun, and the Twiss parameters at the entrance of the monochromator, a conventional electric–magnetic condenser lens is needed^12^.

**Figure 1:**
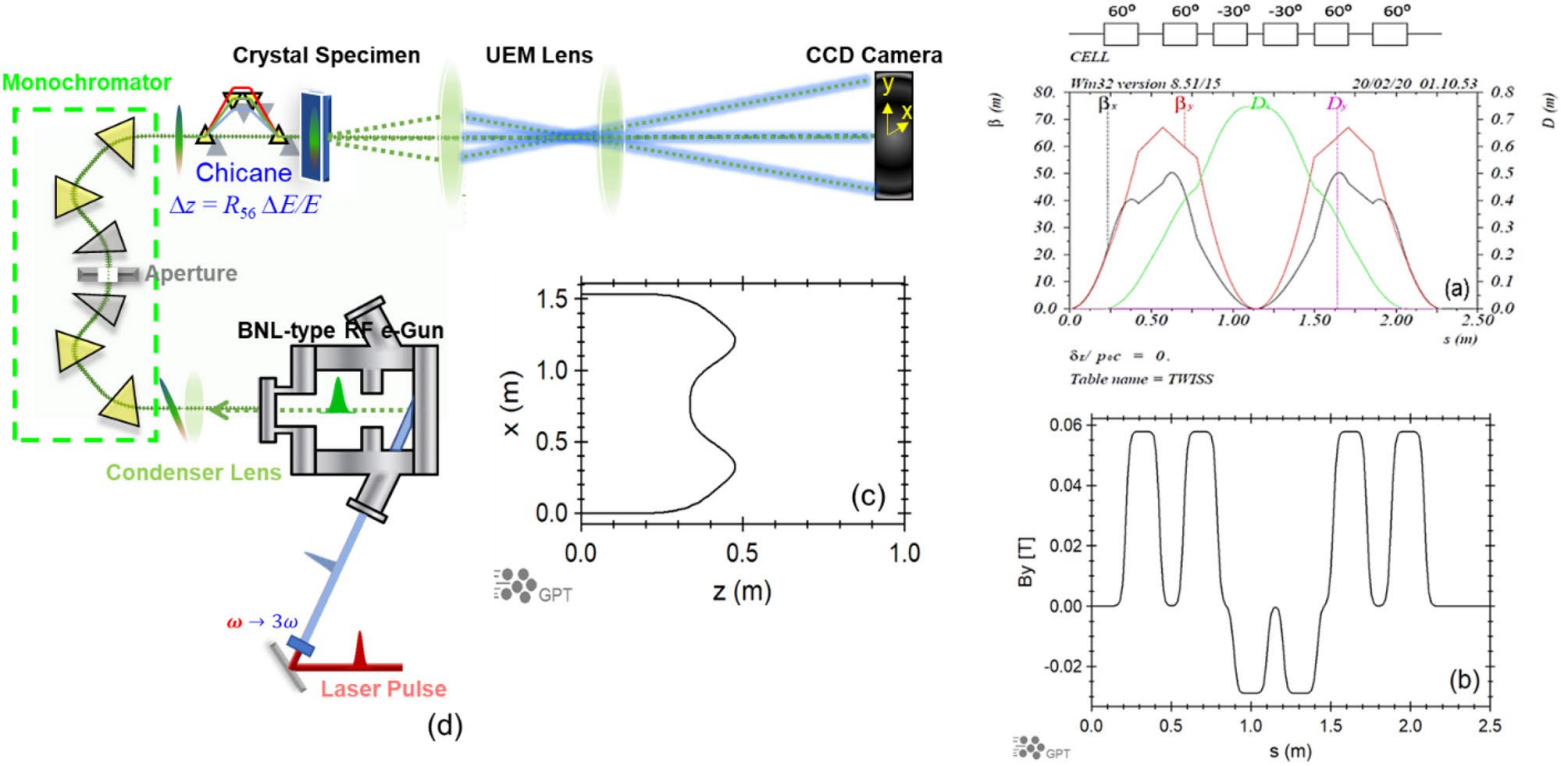
(**a**) The layout and Twiss functions $\beta_{x}$ (black), $\beta_{y}$ (red), $D_{x}$ (green) and $D_{y}$ (magenta). The reverse bends are labeled with the negative sign. The maximum dispersion calculated by MAD code is 0.747 m because the energy spread is defined as $\Delta E{/}pc$ instead of the standard definition $\Delta E{/}E$. It is equivalent to 0.645 m in the standard definition.(**b**) The vertical magnetic field *B*_*y*_. (**c**) The horizontal *x* and longitudinal *z* coordinates in Cartesian system. The vertical coordinate *y* is zero. (**d**) Schematic layout of the entire UEM/UED beamline.

Based on the above considerations, a MeV monochromator, which has four 60° bending magnets with edge focusing (EF) and two 30° reverse bending magnets without EF, is designed to provide the dispersion of 0.645 m. Edge angles of the 60-degree bending magnets are adjusted so that the achromatic condition is achieved at the exit of the monochromator and that the monochromator is a round lens. The use of the reverse bending magnets allows us to adjust *R*_56_ to realize bunch compression. The sequence of magnets and the Twiss functions are shown in Fig. 1a. The total length of the monochromator beam path is $L_{0} = 2.28\,{\text{m}}$, the dipole magnetic field as a function of the longitudinal position *s* in the curvilinear coordinate system describing particle motion in the arc is shown in Fig. 1b. The geometrical layout of the monochromator can be closely resembled by an arc with a 180° bend angle and a 1.5 m diameter, shown in Fig. 1c. The transverse size of the monochromator can be estimated via $2R_{0} = \frac{{2L_{0} }}{\pi } = 2 \cdot \frac{{2.28\;{\text{m}}}}{\pi } \approx 1.5\;{\text{m}}$ which is close to the design value^32^. The magnetic field $B_{y}$, dispersion $D_{x}$, beam size $\sigma_{x}$, azimuthal angle $\theta$, entrance edge angle $\theta_{entr}$ and exit edge angle $\theta_{exit}$ are presented in Table 1 as functions of the longitudinal position $s$. Schematic layout of the whole beamline, from the gun to the detector, is shown as Fig. 1d. The distance from the aperture of the monochromator to the sample is about 1.7 m.

**Table 1:** Monochromator parameters. $D_{x}$ is the horizontal dispersion; *B*_*y*_ is the magnetic field; *θ* is the azimuthal angle in spherical coordinate system; *σ*_*x*_ is the horizontal beam size in RMS assuming $\varepsilon_{x} \approx 10 \;{\text{nm}} \cdot {\text{rad}}$ and $\Delta E/E = 10^{ - 5}$; *θ*_*entr*_ and *θ*_*exit*_ are the entrance and exit edge angles of the monochromator dipole magnets respectively.

Element	s (m)	B_y_ (T)	D_x_ (m)	σ_x_ (µm)	θ (rad)	θ_entr_ (rad)	θ_exit_ (rad)
Begin	0	0	0	4.472	0		
Drift	0.210435	0	0	422.054	0		
B1	0.419875	0.057927	0.087209	762.352	− 1.0472	0.178463	0.315532
Drift	0.569875	0	0.221908	819.436	− 1.0472		
B2	0.779314	0.057927	0.387691	747.840	− 2.0944	0.174533	0.435238
Drift	0.879314	0	0.512028	539.811	− 2.0944		
B3	1.088754	0.028963	0.645	104.315	− 1.5708	0	0
Drift	1.138754	0	0.645	7.849	− 1.5708		
Aperture	1.138754	0	0.645	7.849	− 1.5708		
Drift	1.188754	0	0.645	7.849	− 1.5708		
B3	1.398193	0.028963	0.512028	539.811	− 1.0472	0	0
Drift	1.498193	0	0.387691	747.840	− 1.0472		
B2	1.707633	0.057927	0.221908	819.436	− 2.0944	0.435238	0.174533
Drfit	1.857633	0	0.087209	762.352	− 2.0944		
B1	2.067072	0.057927	0	422.054	− 3.14159	0.315532	0.1784623
Drift	2.277508	0	0	4.472	− 3.14159		
End	2.277508	0	0	4.472	− 3.14159		
Total length = 2.277508				Arc length = 2.277508			

The beta function is 28 m at the entrance of the first magnet and the exit of the last magnet; the beam waist is 21 cm away from this magnet. The beam size is about 4.5 µm at the waist. A condenser lens is required for the matching from the gun to the waist^12^. The maximum dispersion *D*_*x*_ = 0.645 m is in the middle of the monochromator where the aperture is located. The aperture size must be 7.9 µm to achieve the energy spread $\Delta E{/}E = 10^{ - 5}$. To maximize the monochromator efficiency, the beta function has to be < 0.004 m assuming the geometric emittance $\varepsilon_{x} \approx 10 \;{\text{nm}} \cdot {\text{rad}}$.

### Sub-nanometer resolution UEM

#### GPT optimization at 1.6 pC

We aim for the minimization of the energy spread of an electron beam. The monochromator efficiency is defined as the ratio of the number of electrons having energies within the energy acceptance and the total number of electrons. For the high beam charge of 1.6 pC, we numerically maximize the monochromator efficiency via GPT simulation. The important parameters determining the beam energy spread are the laser spot size on cathode ($\sigma_{laser}$), the laser pulse duration ($\tau_{laser}$), the gun phase and the solenoid current. The optimal gun phase can be chosen differently based on a specific application.

**Figure 2:**
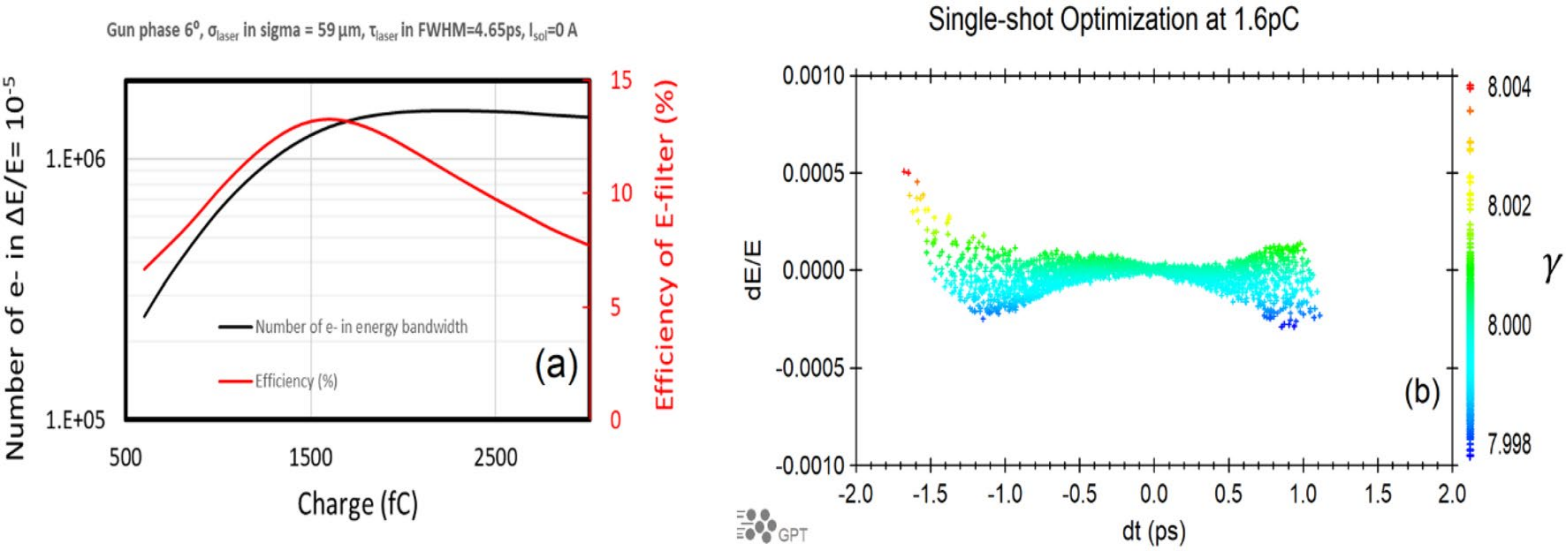
(**a**) (left) The number of electrons (black curve, left vertical axis) within the energy acceptance ($\Delta E{/}E = 10^{ - 5}$) and the monochromator efficiency (red curve, right vertical axis) versus the beam charge. (**b**) (right) Longitudinal phase space of the electron beam at the optimized settings. The positive time in the plot corresponds to the head of the electron beam.

The result of optimization via iteratively scanning those parameters is shown in Fig. 2a. The number of electrons within the energy acceptance (black curve, left vertical axis) and the monochromator efficiency (red curve, right vertical axis) are shown as functions of the beam charge. To optimize the transmission efficiency of the monochromator, one needs to minimize the energy spread via compensating the space charge induced energy chirp by the proper choice of gun parameters, as shown in Fig. 2b. This will be described in detail later. The same time-coordinate convention is adopted throughout the manuscript. The electrons with temporal offsets $dt > 0$ correspond to the bunch head. The pulse duration after the monochromator is about a picosecond. The optimal efficiency of the monochromator can be as high as 13% at the gun phase of 6°, the laser spot size on cathode of $\sigma_{laser} = 59\;\upmu {\text{m}}$, the laser pulse duration (FWHM) of $\tau_{laser} = 4.65\;{\text{ps}}$ and zero solenoid current.

However, for some time-resolved applications, the pulse duration is required to be around a few femtosecond. This can be achieved when the monochromator is configured as “two-color” double pulse setting (see next section). Generally, when shorter pulses are needed, a new set of gun parameters can be found such that *R*_56_ parameter between the cathode and the sample is minimized. Since efficiency will be lower, the charge per pulse out of the cathode needs to be higher. Furthermore, a larger correlation between the electron energy and longitudinal position entails a larger charge density, which leads to a shorter laser pulse and, most likely, to an even higher charge.

### Femtosecond UED

#### Time-resolved single, double and multiple femtosecond pulse UED

In recent years, there is a surge of interest in developing a single-shot time-resolved UED apparatus to visualize lattice dynamic behavior^15,17–24,33,34^. However, in a conventional pump-probe experiment, multiple UED shots with varying time delays after the pump pulse are needed to map out the entire dynamic process mainly due to the limited speed of the detector. The measurement precision is often limited by the timing jitter between the pulses of the laser pump and the UED probe.

To fully benefit from UED as the probe, we propose a novel scheme, enabled by the monochromator, to register a portion of the dynamic process in time by a single-shot time-resolved diffraction pattern via “two-color” or “multi-color” precisely synchronized multiple pulses with adjustable time delays. Since those pulses are originated from the same electron bunch from the photo-cathode RF gun, they will be immune to the shot-to-shot timing jitter. Similar to the generation of two-color x-ray free-electron-laser pulses via a beam with a large energy chirp and a slotted foil^35,36^, we show that, by introducing a time-and-energy correlation (named chirp) to a beam, an aperture with multiple slits at the maximum dispersion of the monochromator can be used to select “multi-color” different energy pulses. After those pulses exit the monochromator simultaneously, a downstream chicane can be applied to independently adjust the time delay between pulses, covering 10 fs up to 1 ps range. Diffraction patterns generated by the “multi-color” pulses are transversely separated on the detector, due to the sufficiently large differences in beam energies. The relative timing jitter among those pulses in a single shot comes only from the magnetic field error of monochromator and downstream chicane magnets fed by highly stable (10^−5^) power supplies, with the perturbation < 10 fs. Furthermore, the optimization of a chirped electron beam to deliver a single femtosecond pulse on the sample is directly applicable to “two-color” and “multi-color” cases. The only difference is to insert an aperture with one slit, two slits and multiple slits, respectively. In the paper, we choose “two-color” double pulses as an example to describe the optimization process.

**Figure 3:**
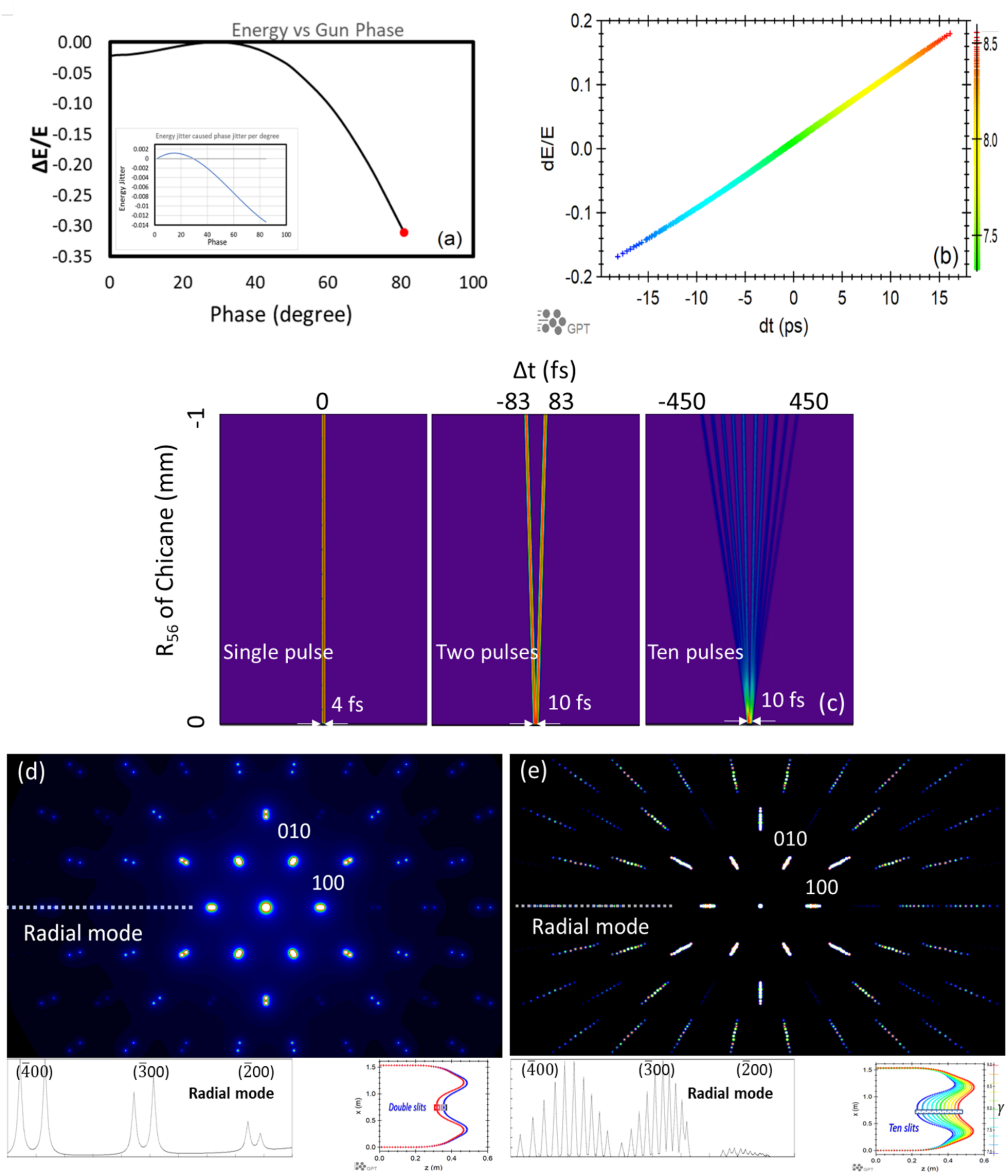
(**a**) The beam centroid energy versus the gun phase. The red dot highlights the optimized phase for the “two-color” case. The shot-to-shot energy jitter per degree of the gun phase jitter is shown in the insert as a function of the gun phase. (**b**) The energy deviation (*dE/E*) versus the time (*dt*) of an electron bunch having 18 pC charge. Electrons with temporal offsets dt > 0 correspond to the bunch head. (**c**) Varying *R*_56_ (vertical axis) of chicane in the range of 0 to − 1 mm, the delay (horizontal axis) in the cases of a single pulse, “two-color” double pulses and “ten-color” ten pulses are shown as the left, middle and right columns, respectively. Each individual pulse has a bunch length of 4 fs and a charge of tens femtocoulomb. (**d**) The bottom left is the diffraction pattern of sample TaS_2_-2H in the “two-color” case. The bottom left insert shows the radial mode covering BD peaks ($\overline{2}00$), ($\overline{3}00$) and ($\overline{4}00$). The bottom right shows the “two-color” slices (blue and red) with ∆*E*/*E* = 5% selected by the double-slits with a transverse separation of 32.25 mm at the maximum dispersion. (**e**) The bottom right is the diffraction pattern of sample TaS_2_-2H in the “ten-color” case with the energy separation of 3%. The bottom left insert shows the radial mode covering BD peaks ($\overline{2}00$), ($\overline{3}00$) and ($\overline{4}00$). The bottom right shows the “ten-color” slices with 3% energy separation selected by the ten-slits at the maximum dispersion.

Two electron bunches with different beam energies (e.g. 5% difference) can be generated by the space charge effect in the high charge (> 10 pC) case^10,11^, together with the proper choice of the accelerating gradient $dV{/}dt$ controlled by the gun phase, which is essential for achieving the maximum energy chirp. The energy chirp induced by the accelerating gradient has the same sign with the energy chirp caused by the space charge effect at the charge of 18 pC, the gun phase of 80°, the laser spot size on cathode of $20\;\upmu {\text{m}}$, the laser pulse duration (FWHM) of 8 ps and the solenoid current of 68 A. The beam centroid energy as a function of the gun phase is shown in Fig. 3a, the optimized gun phase is highlighted by the red dot. An up to 30% peak-to-peak energy difference can be generated by this method, as shown in Fig. 3b. To maintain the same beam energy, the accelerating voltage is constantly adjusted according to the different gun phase setting. In the present monochromator design, the energy chirp carried by the beam is optimized with the minimum time jitter between these “two-color” beam slices to achieve the achromatic condition. There is no need of a 4th harmonic cavity to adjust the energy chirp. This greatly simplifies the operational condition.

Depending on the purpose of optimization, the relationship between the beam energy and the gun phase (Fig. 3a) provides a wide range of flexibilities. Unlike the “two-color” case, to optimize the transmission efficiency of the monochromator, one needs to minimize the energy spread via compensating the space-charge induced energy chirp. A proper choice of the gun phase provides the right value of the accelerating gradient with the opposite sign of the space charge effect to cancel out the space-charge induced energy chirp. For example, the optimized gun phase is 6° for the beam charge of 1.6 pC. However, it is important to set the gun phase at the flat top of 30° when the accumulation mode is used for UED/UEM since in most cases the shot-to-shot energy jitter is dominated by the gun phase fluctuation. Even in the worst case, the phase jitter has the minimum impact on the accumulated energy spread of the electrons because of the zero phase-to-energy conversion coefficient, shown as the insert of Fig. 3a. The drawback is that at the fixed gun phase of 30° after re-optimization, the single-shot electron flux within the energy acceptance of the monochromator will be degraded by about 25% and 20% in the high-charge (1.6 pC) and low-charge (50 fC) cases, respectively. Here, 0.1° phase and 2∙10^−4^ amplitude jitters in FWHM of the RF field in the photo-cathode gun are considered in the simulation.

The current design of the monochromator is optimized with $R_{56} = 0.069 \;{\text{m}}$ compensating the energy chirp (Fig. 3b) to satisfy the achromatic condition and remove the time jitter caused by different beam energies. It is worth noting that the value of $R_{56}$ chosen here is somewhat arbitrary and can be easily changed to accommodate the need of the system. For instance, two beam slices with a 5% energy separation (the “two-color” case) can be selected by a double slit installed in the maximum dispersion area of the monochromator^35,36^. The dispersion of 0.645 m and the 5% energy separation determine the 32.25 mm transverse distance between those two slits. The delay between these “two-color” double pulses can be independently adjusted via a chicane downstream of the monochromator with the limit of $R_{56} = - 1 \;{\text{mm}}$. Besides, the chicane can be used to compensate the residual energy chirp after the monochromator, maintaining the achromatic condition for a broad range of the electron beams with minimum timing jitter.

Each slice of the “two-color” double pulses has the charge of tens femtocoulomb and the pulse duration a few femtoseconds. Since most of the charges (≥ 99.5%) are lost at the aperture of the monochromator, it is numerically evident via GPT simulation that space-charge induced energy spread and bunch lengthening are negligible. One can probe structural dynamics with a 10 fs time resolution using two pulses with a tunable delay ranging from 10 to 160 fs^37^. These two pulses are separated on the detector transversely due to sufficiently different electron beam energies. Besides, the “two-color” double pulse scheme is easily expandable to “multi-color” multiple pulse schemes. Eventually, the maximum number of “multi-color” pulses is limited by the minimum energy separation, which is still detectable by the detector, e.g. ≥ 3%. This number can be as big as ten. By varying the energy-dependent path length difference *R*_56_ of the chicane in the range of 0 to − 1 mm, the delay between the “two-color” double pulses can be varied from 10 to 166.7 fs, shown as the middle column in Fig. 3c. The minimum delay of 10 fs is limited by the precision of magnet power supplies. Similarly, the maximum delay of “ten-color” ten pulses can be adjusted to cover the dynamic range up to 900 fs, shown as the right column in Fig. 3c. The diffraction patterns of the TaS_2_-2H sample in the “two-color” case with the energy separation of 5% and in the “ten-color” case with the energy separation of 3% are shown in Fig. 3d,e, respectively.

A similar line-intensity profile of the diffraction pattern, covering BD peaks ($\overline{2}$00) ($\overline{3}$00) and ($\overline{4}$00), is shown as the bottom-left insert in Fig. 3d,e. As a proof-of-concept, those BD peaks formed by electrons with different energies are well separated in the diffraction pattern in both the “two-color” and “ten-color” cases. Therefore, a “multi-color” scheme with a tunable delay between the pulses can potentially provide a single time-resolved diffraction image for visualizing lattice dynamic behavior.

### Diagnosis of energy spread and divergence

Based on different peak-broadening features (transverse and radial) due to different electron beam parameters (divergence and energy spread), one can establish the one-to-one mapping between the electron beam parameter and the induced peak broadening. From such unique mapping, the beam energy spread and divergence can be extracted from the radial and transverse modes of the BD peak broadening, respectively. Applying the monochromator, one can experimentally vary the radial peak-broadening mode by changing the bandwidth of the monochromator therefore calibrating the measured energy spread of the electron beam.

**Figure 4:**
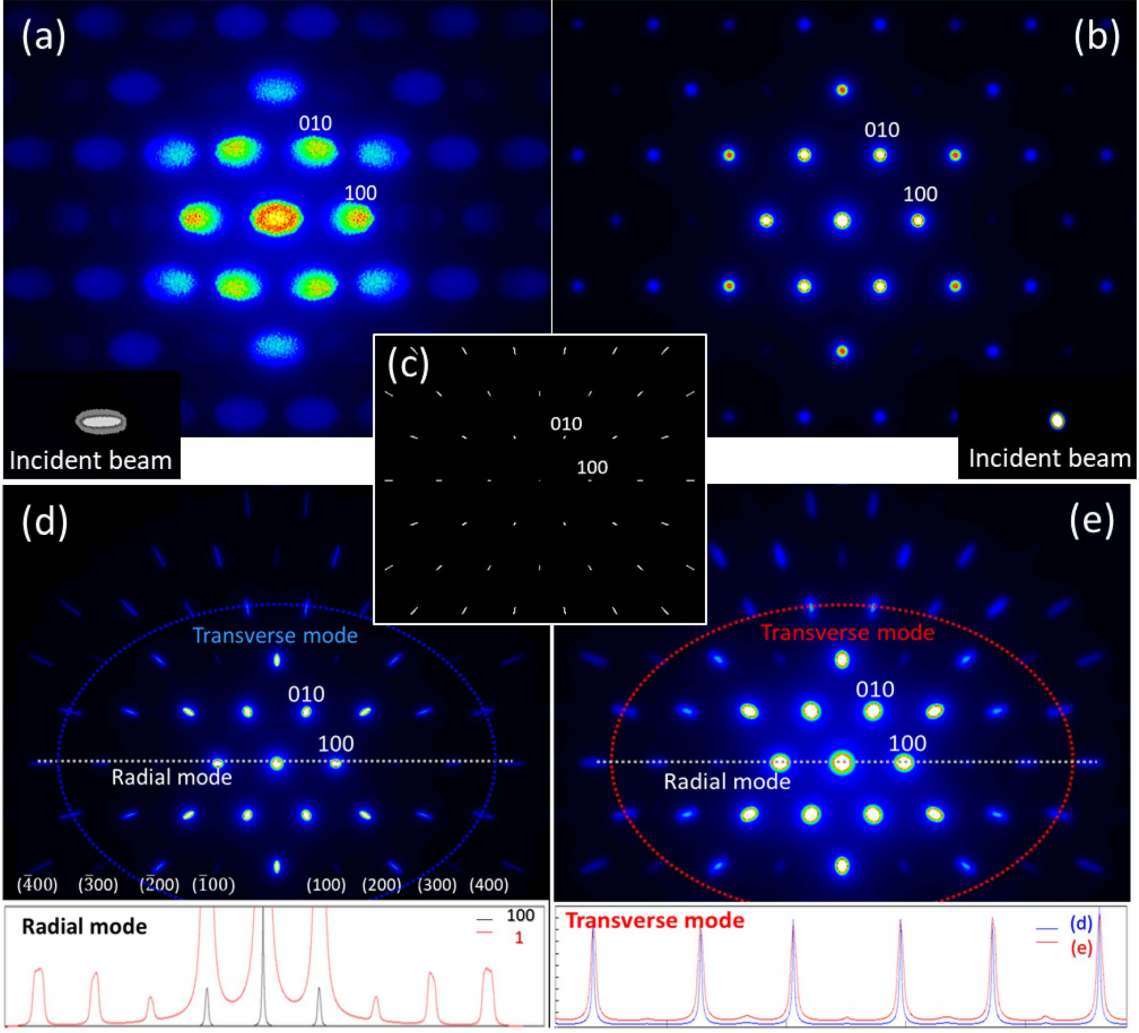
(**a**) The (001)* diffraction pattern of a TaS_2_-2H sample. The electron beam is shown as the bottom-left insert; the horizontal and vertical FWHM angular divergences are 0.5 mrad and 0.3 mrad, respectively. (**b**) The same diffraction pattern as of (**a**). The electron beam is shown as the bottom-right insert; the divergence is 0.1 mrad in both directions. (**c**) The diffraction pattern of TaS_2_-2H sample. The beam with zero divergence in both directions but with an energy spread of $\Delta E{/}E = 5\%$, without considering the PSF effect. (**d**) The only difference with (**c**) is: applying the instrumental PSF via Lorentz convolution with the width of 0.04 mrad, the diffraction pattern is close to the experimental observation. The insert is the horizontal line-intensity profile, representing the radial mode, with the full scale of maximum intensity (black) and zoomed 1% of the full scale (red). (**e**) The diffraction pattern from electrons with 0.1 mrad divergence and 5% energy spread. The line-intensity profiles, projected around the blue and red ellipses with respect to (**d**) and (**e**) respectively, are shown as the blue and red curves of the bottom insert.

We did the proof-of-principle simulations using the TaS_2_-2H sample. The electron diffraction patterns (EDP) are calculated with our own computer code, which has been used for quantitatively determining crystal structure and charge distributions of crystals^37–39^. The reflection intensities are calculated based on the Bloch wave method, which takes dynamical effects in electron diffraction into a full consideration. The diffraction intensity depends on the sample thickness, the incident beam direction and the beam energy, etc. The first simulation is performed using the TaS_2_-2H sample probed by an electron beam with the FWHM angular divergence of 0.5 mrad and 0.3 mrad in the horizontal and vertical directions, respectively. The electron beam is represented by hundreds of macroparticles^40^. The worst-case scenario was chosen for proof-of-concept purposes. Since it is impractical to treat each electron as an individual particle in simulation, e.g. 1.1 ⋅ 10^8^ particles are needed for 18 pC, an electron beam distribution is often viewed as a collection of macroparticles. To ensure that macroparticle simulation code includes the most important physics effects^40^, each macroparticle in our case represents thousands of electrons or more. There exists an important feature—the divergence of an incoming electron beam broadens BD peak widths similarly among all the peaks with different Miller indexes (*h k l*), obeying the Bragg’s law $2d_{h,k,l} \sin \theta = n \cdot \lambda ,\;{\text{where}}$ *d*_*h,k,l*_ equals to *G*^−1^ and *G* is the reciprocal lattice vector. Therefore, the common part of the peak-broadening with respect to different Miller indexes can be used to extract the x and y angular divergences of an electron beam. A diffraction pattern generated by electrons diffracted from a TaS_2_-2H sample is shown in Fig. 4a. The 2D profile of the incident beam is shown in the bottom-left insert. All the peaks with different Miller indexes are broadened in a similar way determined by the divergence of the electron beam. A condenser lens with two sets of the quadrupole multiplets can be applied to place the waist of the beam at the sample and simultaneously adjust the spot size to minimize the divergence^10^. Optimization of the beam divergence to 0.1 mrad via the quadrupole lens focusing in the simulation results in a great improvement of the diffraction pattern with much sharper BD peaks, shown in Fig. 4b. We call such peak broadening, clearly measurable in the diffraction pattern, as the transverse peak-broadening mode.

The second simulation is performed using the TaS_2_-2H sample probed by an electron beam with zero divergences but with the energy spread of $\Delta E{/}E = 5\%$. The result is shown in Fig. 4c without considering the point-spread function (PSF) of the detector. Ideally, each peak formed by a single electron diffracted by TaS_2_-2H appears as a geometrical point in the diffraction pattern. In the real experiment, the finite pixel size, a spread of the signal, noisy background and other instrumental factors contribute to the PSF^10,11^. To reproduce the BD peak broadening obtained in the experiment, we applied the PSF with the width of 0.04 mrad via Lorentz convolution. A similar PSF has been applied to all diffraction patterns, except to one shown in Fig. 4c to show the detector PSF effect.

The difference in the electron beam energy corresponds to the difference in the De Broglie wavelength *λ* via $\Delta E{/}E = - \Delta \lambda {/}\lambda$. The BD peak broadening caused by the energy spread is proportional to the Bragg reflection, or scattering vector, order *n *via $\Delta \theta_{h,k,l,n } \approx n \cdot \Delta \lambda /\left( {2 \cdot d_{h,k,l} } \right)$. Therefore, we define this energy-spread induced peak broadening as the mode of radial peak broadening. As it was experimentally demonstrated, sharp diffraction patterns with a good signal-to-noise ratio can be obtained in a single shot with high-order ($6\overline{6}0$) BD peak at $\theta_{{{\text{Bragg}}}} \approx 6.26\;{\text{mrad}}$ in a single-crystalline Si^41^. We can potentially extract the radial peak-broadening component from a diffraction pattern and obtain the beam energy spread information. The insert in Fig. 4d shows the horizontal line-intensity profile with the full intensity scale (black) and with zoomed 1% of the full scale (red). The radial peak-broadening is clearly measurable. This also explains why we look at the BD peaks with Miller index larger than (100) for the sufficient color separation in the “multi-color” scheme.

Figure 4b,d show the transverse-only and radial-only modes caused by the beam divergence and energy spread, respectively. In the experiment, an electron beam has both the divergence and energy spread and the diffraction pattern is a convolution of both transverse and radial modes. The diffraction from an electron beam with 0.1mrad divergence and 5% energy spread is shown in Fig. 4e. The transverse mode can be examined via the line-intensity profile along the ellipse shown in Fig. 4d,e, and the results are shown as the insert in Fig. 4e as the blue and red curves, respectively. The non-zero width of the blue curve is due to the PSF and the difference between red and blue curves is caused by the beam divergence.

It is numerically evident that single-shot diffraction imaging can be used to characterize the energy spread and angular divergence of the electron beam. The combination of a single-shot decomposition of the electron beam energy spread and divergence with the shot-to-shot spatial and energy jitter analysis, as experimentally demonstrated before^42^, can provide comprehensive information of the electron beam, including the energy spread, divergence, shot-to-shot spatial pointing and energy jitters.

## Discussion

A unique MeV monochromator with the 10^−5^ energy resolution and optimized *R*_56_ to preserve bunch length and reduce timing jitter has been designed. It can be applied to minimize the energy spread in the single-shot mode and the shot-to-shot energy jitter in the accumulation mode demanded by high electron flux applications. We numerically optimized the electron transmission efficiency of the monochromator in the high-charge (1.6 pC) and low-charge (50 fC) cases with the maximum efficiencies of 13% and 25%, respectively. The number of electrons transmitted by the monochromator can be as high as 1.3 million per pulse. In the accumulation mode, the degradation of single-shot flux is minimized with the proper choice of gun phase and re-optimization. Despite the dominating shot-to-shot jitter of the RF phase, the electron flux is nearly proportional to the repetition rate. The result provides a major step forward, towards the UEM with a sub-nm resolution and the time resolved UED with an angular resolution comparable to XFEL (≤ 10^−3^). A MeV electron beam with a femtosecond bunch length has been demonstrated experimentally^30^. Thanks to the reverse-bend achromat design of the monochromator, the energy-dependent timing jitter can be greatly reduced with no need of quadrupoles. This makes the manufacturing, commissioning, switching between different operational modes and daily tuning easier. The “two-color” double pulses enabled by the monochromator with a tunable delay in the sub-picosecond range via a magnetic chicane and a femtosecond pulse duration, can potentially provide the time resolution required by pump-probe experiments studying structure dynamics.

Based on peak-broadening features (transverse and radial) caused by different electron beam parameters (divergence and energy spread), one can apply the one-to-one mapping for the noninvasive diagnosis of energy spread and divergence of the electron beam. This method, together with the shot-to-shot spatial and energy jitter measurement experimentally demonstrated before^37^, can provide comprehensive information of the electron beam.

Furthermore, the monochromator based on electromagnetic dipole magnets provides the energy-scan capability. Such an energy scan with narrow bandwidth can provide real-time energy matching for UEM (especially for the UEM systems based on the permanent magnets), the on-momentum flux maximizing for UED and energy measuring with an improved energy resolution for energy-loss spectroscopy.

## Methods

### Design of the monochromator

A MeV monochromator with 10^−5^ energy resolution is designed to achieve achromaticity in a broad range of UEM/UED operation. Six electromagnetic dipoles (four 60° bends and two − 30° reverse bends) provide the required dispersion of 0.645 m. The edge angles of the 60-degree bending magnets are adjusted such that an achromatic condition is achieved at the exit of the monochromator and that the monochromator is a round lens. The overall energy-dependent path length difference $R_{56}$ between the cathode and the sample is minimized^30^. The length of the electron bunches and the energy-dependent timing jitter can be greatly reduced down to the 10 fs level.

Since the maximum dispersion (0.645 m) of the monochromator provides a sufficiently large transverse separation for the incoming energy-chirped electron beam, an aperture with multiple slits can be applied to select multiple beam slices (2 to 10) with different energies. Thanks to the achromat design, those “multi-color” pulses exit the monochromator simultaneously. Therefore, the time delay between those pulses can be independently adjusted via a downstream chicane, covering the time duration 10–900 fs. Each individual pulse has the charge of tens fC and the pulse duration of a few fs. Diffraction patterns generated by the “multi-color” pulses are transversely separated on the detector, due to the sufficiently large differences in beam energies (≥ 3%). There is no need for any RF deflector. Furthermore, one can experimentally vary the radial peak-broadening mode by changing the bandwidth of the monochromator via inserting a wedge aperture, therefore calibrating the energy spread measurement.

### Calculations of electron diffraction patterns

An incoming electron beam to the sample is represented by a collection of macroparticles in GPT simulation^31,40^. Each macroparticle has the coordinate $nmacro_{i}$, where *i* = 1, 2 to *N* and *N* is the total number of macroparticles in the beam. The charge of a beam is determined by $C = \mathop \sum \limits_{i = 1}^{N} c_{i} = \mathop \sum \limits_{i = 1}^{N} nmacro_{i} \cdot q$, where *q* is the charge of an electron. The reflection intensities are calculated based on the Bloch wave method for each individual macroparticle^37–39^, and the reflection intensities are scaled by the charge of each macroparticle. The diffraction pattern is obtained by the summation of all reflection intensities from all macroparticles.
